# Experimental Studies on Thermal Oxidation and Laser Ignition Properties of Al-Mg-Li Powders

**DOI:** 10.3390/ma16216931

**Published:** 2023-10-28

**Authors:** Yingying Lu, Kai Ma, Changchao Guo, Ming Jiang, Chengfeng Wu, Shipeng Li, Shaoqing Hu

**Affiliations:** 1 School of Aerospace Engineering, Beijing Institute of Technology, Beijing 100081, China; 2 Xi’an Modern Chemistry Research Institute, Xi’an 710065, Chinawuchengfeng@tju.edu.cn (C.W.)

**Keywords:** powder ramjet, Al-Mg-Li alloy powders, laser ignition, ignition delay time

## Abstract

Powder ramjets are a kind of vehicle propulsion system with high specific impulse and efficiency. They provide significant benefits in terms of extended propulsion and thrust adjustment. The pursuit of a highly reactive fuel appropriate for powder ramjets is likely to stimulate advancements in innovative propulsion systems, which are crucial for deep space exploration and long-term space missions. This work presents experimental studies on the thermal oxidation and laser ignition performance of aluminum–magnesium–lithium powders at atmospheric pressure. TG-DSC curves of powders in three heating rates were obtained. The ignition processes and ignition delay times were recorded by a CO_2_ laser ignition experiment system at a laser power of 10~60 W. The results show that at a lower heating rate of 10 K/min, the powder’s thermal hysteresis is less, and the powder energy released in stage I is more concentrated. However, the degree of heat release concentration approached a similar level at heating rates of 30 K and 50 K. The ignition delay time decreased as the laser flux density increased. When the laser flux density exceeds 80 W/cm^2^, the effect of laser power on the ignition delay time decreases. At atmospheric pressure, the mathematical relationship between ignition delay time and laser flux density is given. Finally, the powder ignition processes at different laser powers are represented graphically.

## 1. Introduction

The powder ramjet compresses ambient air at high flight speeds via the intake, enabling the fuel to combine with the air and burn at high flow speeds, resulting in a high specific impulse. The ramjet system is simple and there is no oxidizer in the fuel. As a result, it is expected to be a more cost-effective propulsion system choice than traditional solid rocket propulsion systems. However, the powder ramjet requires the vehicle to reach a certain flight speed before it can be activated and the residence time of the powder fuel in the combustion chamber is very short when the flight speed is too high. Therefore, the powder ramjet engine fuel must have both high energy density and be easy to ignite. Some highly active metal powders are obvious candidates, such as Al, Mg, B, etc. [1,2].

Al is a prospective energetic fuel that exhibits a considerable advantage due to its high energy, safe byproducts, and low preparation cost. It is frequently utilized as a high-energy propellant component [3,4,5,6,7]. Al has undergone substantial research as a powder fuel for new propulsion systems with the development of powder ramjet technology [8,9,10,11,12,13]. According to recent studies, the oxide layers in industrial Al powder are typically several nanometers thick. The internal aluminum core will not ignite or burn properly because the aluminum oxide film’s melting point (2054 °C) is substantially greater than that of aluminum (660 °C). Agglomerating around the new Al droplets in the form of oxide caps [14,15,16,17], the oxide film created by the oxidation reaction of Al powder during the combustion process will also prevent additional Al oxidation from occurring [18]. In order to improve the ignition and combustion characteristics of Al, researchers have found a way to prepare an alloy by adding a low boiling point metal to prevent the oxide cap from agglomerating on the droplet surface and improve the reaction of Al through the vaporization of the low boiling point metal, such as Mg [19,20]. Mg is a potential metal fuel with high energy density and low ignition temperature. The boiling point of Mg (~1090 °C) is significantly lower than that of Al (~2519 °C). However, it was discovered that the reaction only results in the selective oxidation of Mg in the Al-Mg alloy, and does not promote oxidation of aluminum at lower temperatures. Finding a substance that can encourage the oxidation of Al to create a ternary alloy is a potential strategy as the igniting property of Al-Mg alloy powder does not perform as expected. The active metal Li has a high mass calorific value of 45.8 kJ/g. The addition of Li to an Al-Mg alloy is more likely to lower the ignition initiation threshold and improve the ignition characteristics of the alloy powder because Li has a lower ignition temperature and stronger oxidation reaction activity than Al and Mg [21,22,23]. Additionally, the boiling points of Li (1342 °C) and Mg (1090 °C) are significantly lower than aluminum (2519 °C). An increase in the proportion of boiling vaporization within the alloy during the ignition and heating of the alloy powder can speed up the powder’s reaction rate and increase the efficiency of the release of combustion energy from Al-based powder fuels [24,25].

Many studies have demonstrated the feasibility of Al-based alloys to improve combustion performance compared to pure Al powders, with the majority of studies concentrating on Al-Mg, Al-W, or Al-Li [26,27,28,29,30,31,32,33,34,35]. To obtain the ignition characteristics of alloy particles, Corcoran et al. [36] studied single particles of the Al-Mg alloy (1–50 µm, Mg:10–53% mol) in high-temperature mixed acetylene/airflow. It was found that the combustion time of the alloy particles can be expressed in the form of *t* = *a* × *d^n^* with a time index *n* of 0.6–1.0. In the preceding expression, the symbol ‘*t*’ denotes the combustion time of the alloy particle, ‘*d*’ signifies the pellet diameter, while ‘*a*’ and ‘*n*’ stand for dimensionless coefficients. The combustion characteristics of five Al-Li alloys with different component ratios were studied by J. T. MOORE in an oxygen/argon combination or a water vapor atmosphere [34]. The findings demonstrate that the element Li can encourage the melting and low-temperature oxidation of aluminum, and that the reaction products of the Li-O_2_ interaction do not obstruct aluminum oxidation, increasing aluminum’s reaction efficiency. However, the storage safety and stability of Al-Li alloy powders are reduced when the Li content reaches 10% or more. In addition, Li has a lower density than Al and Mg. By adding an element such as Mg, which has a higher volumetric calorific value than Li, the material’s application area can be expanded. Al, Mg, and Li are all metallic materials that can react with air and water, which confers significant advantages in the development of air and water ramjet engines [8,37]. Therefore, ternary alloy and Al-Mg-Li powders appear to be useful research areas for novel materials, taking into account the materials’ combustion efficiency, safety, and application potential. Investigating the thermal oxidation and ignition characteristics of the Al-Mg-Li alloy powders is crucial for a deeper understanding of the potential applications of this material.

According to the above description, Al-Mg-Li alloy powders need to be studied for the purpose of understanding their potential applications. Therefore, this paper focuses on three questions: (1) the thermal oxidation behavior of Al-Mg-Li powders when subjected to slow hot-air heating in an air atmosphere; (2) the ignition and combustion characteristics of Al-Mg-Li powders under large heat flow density heating conditions; and (3) the ignition and combustion mode of particles in powders. To answer the three questions above, thermogravimetric analysis was performed to obtain the initial oxidation temperature and energy release characteristics of the powder samples (corresponding to the first question). Further, ignition tests at different laser powers were used to investigate the correlation law between ignition delay times and laser heat flux density of Al-Mg-Li powders (aiming to answer the second question). Additionally, the combustion condensed products (CCPs) of the laser ignition tests were characterized by SEM and EDS, which showed the morphology and components of the products clearly. This section illustrated the combustion pattern of Al-Mg-Li powders during the laser ignition process and is related to the third question proposed above. Finally, the powder evolution mechanism at different laser powers is illustrated according to the ignition snapshots and SEM images of the condensed products. This study provides data on the ignition characteristics of Al-Mg-Li alloy powders, which is significant for the screening and application of new fuels used in ramjets. 

## 2. Materials and Methods

### 2.1. Particle Sizing and Imaging

**Figure 1:**
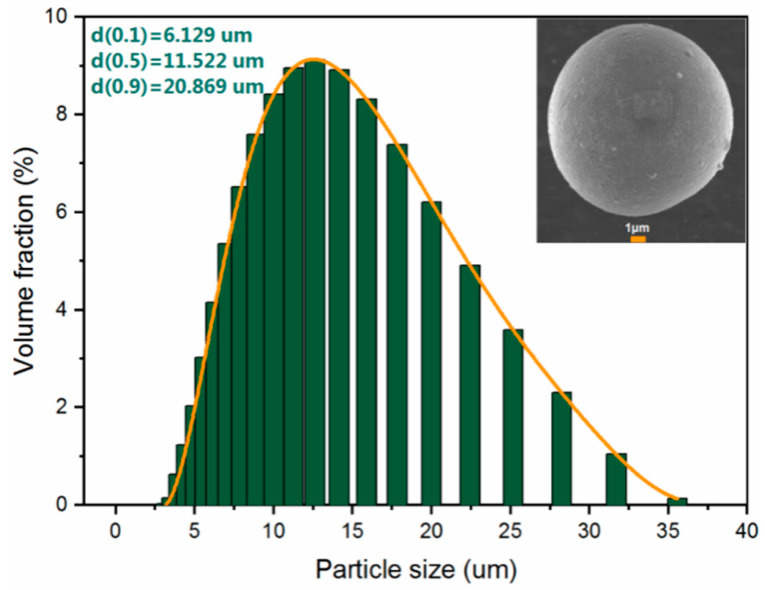
The size distribution of Al-Mg-Li powders.

**Figure 2:**
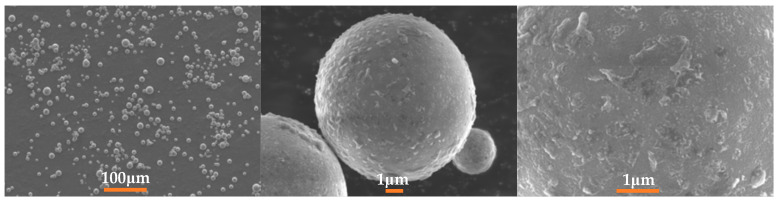
SEM images of the sample (Al-Mg-Li powders).

**Figure 3:**
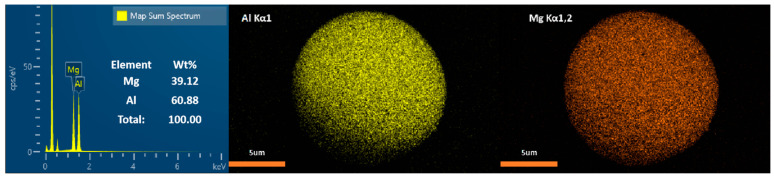
EDS images of the sample (Al-Mg-Li powders).

Al-Mg-Li powders were prepared by the Huazhong University of Science and Technology (Wuhan, China) via a close-coupled gas atomization method. Drawing upon the insights derived from prior investigations into the ignition and combustion characteristics of Al-Mg alloys with varying component ratios [38], and with due consideration to the density characteristics of these alloys, we have established the specific Al/Mg/Li component ratios for our current research endeavor. The raw material feeding mass ratio of Al:Mg:Li is 6:3:1. In addition, the measured values are listed in Table 1. The mass ratios of Al and Mg were obtained by the titration method. The mass fraction of Li was determined by the Inductively Coupled Plasma Atomic Emission Spectrometer (ICP-AES) method. In the ICP-AES methodology, the instrumental apparatus employed is the Agilent 5110 ICP-OES, which is manufactured by Agilent Technologies, headquartered in Santa Clara, California, USA.Samples used in this work were screened by a sieve of 500 mesh. The laser particle size analysis method was used to analyze the size distribution of samples and the test equipment model number was the Mastersizer 2000, manufactured by Malvern Panalytical, Malvern, UK. Size analysis results are provided in Figure 1. The morphology, energy-dispersive X-ray spectroscopy (EDS), and elemental mapping measurements were performed using the Oxford 51-XMX with an SEM instrument, manufactured by Oxford Instruments, Oxford, UK. SEM and EDS images of the powders are depicted in Figure 2 and Figure 3, respectively.

### 2.2. TG-DSC Characterization

To investigate the effects of different ambient heating rates on the thermal oxidation characteristics of the powder samples, 6 mg of samples were placed in an alumina crucible and subjected to TG-DSC simultaneous thermal analysis under an air atmosphere with an airflow rate of 50 mL/min. The heating rates were 10 K/min, 30 K/min, and 50 K/min, respectively. The test temperatures were increased from 30 °C to 1300 °C. The experimental equipment used was an STA-449F5 synchronous thermal analyzer, NETZSCH, Waldkraiburg, Germany.

### 2.3. Laser Ignition System

**Figure 4:**
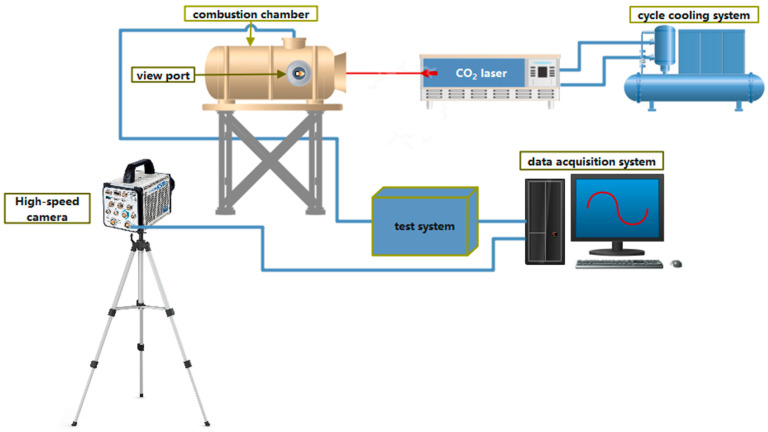
The laser ignition experimental system.

**Figure 5:**
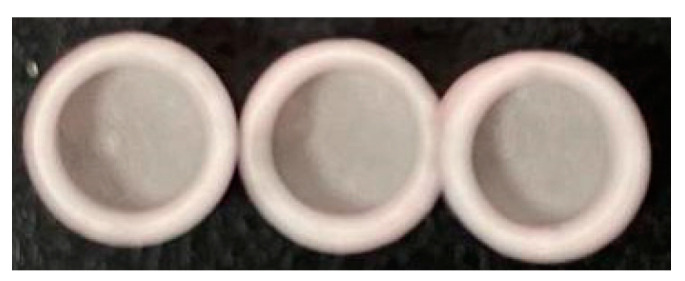
Experimental samples.

The laser ignition system is mainly composed of five parts: a laser energy system, combustion chamber, cycle cooling system, high-speed camera, and data acquisition system. The laser source is a CO_2_ continuous laser (SLC110) with a maximum power of 120 W and output wavelength of 10.6 µm, 1000 Hz. In order to safeguard the integrity of the container during powder ignition, an aluminum oxide crucible is employed as the receptacle. The inner chamber of this crucible features dimensions measuring 5 mm in diameter and 4 mm in depth. Additionally, the laser spot diameter has been set to 5 mm to correspond with the inner cavity dimensions of the crucible. The mass of the powder sample used for the experiment was 20 mg. Laser powers ranged from 10 to 60 W, resulting in corresponding laser flux densities within the range of 50.96 to 305.73 W/cm^2^. The determination of laser flux density is achieved by dividing the laser power by the effective area of the laser spot on the test specimen, specifically, the internal area of the crucible. The irradiation power was measured with a laser power meter (Ophir Nova II, error 2%). During the ignition process, the duration of the laser is 2 s. Further, the position *t*_0_ at which the ignition signal begins and 90% of the signal maximum *t*_0.9_ are two common values that are used to describe the ignition delay time (*t*_ig_) of the sample: *t*_ig_ = *t*_0.9_ − *t*_0_ [39]. Due to the near proximity of the two, the time between the laser beam starting and reaching the sample surface is ignored in this instance. The combustion chamber has a glass viewport to observe the ignition process inside. A Canon high-speed camera is placed in front of the combustion chamber window to record the combustion flame at 1000 frames/s during the ignition process. The data acquisition system consists of a TEKDP04034 high-performance digital oscilloscope, a desktop computer, and an optoelectronic test circuit, which are used for testing, recording, and processing the experimental parameters. The experimental system model is shown in Figure 4, and the test samples are shown in Figure 5.

## 3. Results and Discussion

### 3.1. Oxidation Procedures in Different Heating Rates

**Figure 6:**
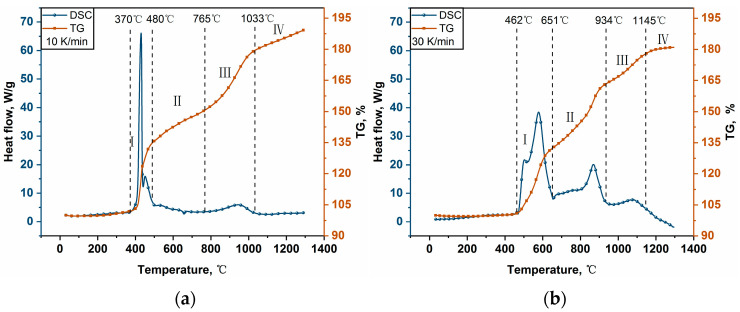

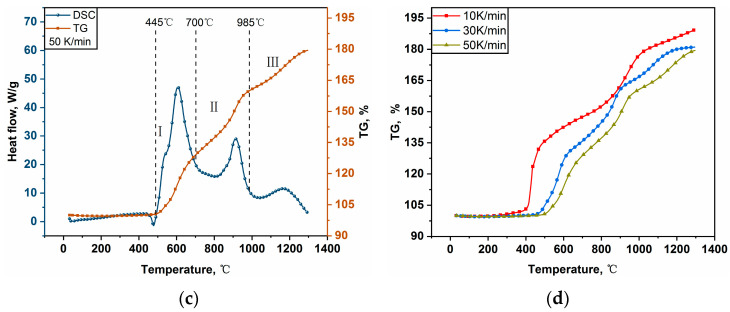
TG-DSC curves Al-Mg-Li alloy at different heating rates in air. (**a**) Curves at a heating rate of 10 K/min; (**b**) curves at a heating rate of 30 K/min; (**c**) curves at a heating rate of 50 K/min; (**d**) comprehensive comparison of TG curves at different heating rates.

Thermal oxidation characterization in three heating rates of 10, 30, and 50 K/min was performed by combined TG-DSC experiments. The powder samples’ weight gain trajectories and heat change curves are shown in Figure 6a–d. The results of the TG-DSC experiments were classified into different stages according to the change in the slope of the TG curves, as shown in Figure 6a–c.

Figure 6a,b demonstrates that the oxidization of Al-Mg-Li can be roughly divided into four stages according to the rate of mass increase. The four stage cut-off temperatures in Figure 6a are 370~480 °C, 480~765 °C, 765~1033 °C, and 1033~1300 °C, respectively. It can be seen from the DSC curve in Figure 6a that two adjacent exothermic peaks arise in the temperature range of 370~480 °C, which should be related to the exothermic oxidation of Li and Mg. The Al-Mg-Li alloy used in this work exhibits a lower initial oxidation reaction temperature than the Al-Mg_0.29_ alloy [40]. The sample weight increase is about 132% in this stage. In stage II, there was a weak heat absorption peak in 480~765 °C corresponding to a temperature of about 650 °C, which is the melting temperature of the Al-Mg alloy. In this temperature interval, the sample was slowly oxidized, which was accompanied by a small amount of heat absorption by the melting of the Al-Mg alloy, and no obvious oxidation exothermic peak was seen. In stage III, the DSC curve showed an exothermic peak near 950 °C. This is attributed to the continuous increase in the sample temperature and the melting of the particle core. Aluminum-based active metal components oxides with heat release. As a result, the DSC curve shows a wide range of exothermic peaks. In addition, the weight gain of the TG curve at this stage was significantly accelerated.

In contrast to Figure 6a, the temperature interval in Figure 6b where the starting oxidative exothermic peak is located is significantly shifted back. Furthermore, the width of stage I is greater than in Figure 6a. The sample exhibits a broad exothermic peak in stage II, associated with a temperature interval of 651~934 °C. Stage II is based on the simultaneous melting and oxidation of Al-Mg alloy, and then the synergistic effect of melting heat absorption and oxidation exotherm slows the overall rise in ambient temperature. As the powders become molten, a noticeable exothermic peak (about 880 °C) develops due to a significant increase in the rate of oxidation. Furthermore, the DSC curve retains a faint exothermic peak in stage III, corresponding to the oxidation exotherm of the remaining active Al.

The thermal oxidation curve in Figure 6c is similar to that in Figure 6b. The initial exothermic peak occurs at a higher temperature range than in Figure 6a, and the DSC curve also contains three exothermic peaks. Furthermore, it can be seen in Figure 6c that there is a weak heat absorption in the temperature range of 400~500 °C. According to the data on the melting points of Al (660 °C), Mg (649 °C), and Li (180.5 °C), it is evident that this phenomenon cannot be attributed to the melting of Li. By considering the melting points of Al and Mg, we tentatively infer that the observed heat absorption peak results from the partial melting of Al and Mg. This assertion finds support in the findings reported by Aly Y. [27] concerning Al-Mg alloys. Dreizin concluded that the heat absorption peak at 450 °C corresponds to the eutectic melting. Some of the samples we used have a portion of Al-Mg eutectic, which exhibits a significant heat absorption peak on the TG curve. In stage I (445~700 °C), a vigorous oxidation reaction initiates Li and Mg, resulting in the release of a substantial amount of heat. In stage II (700~985 °C), simultaneous melting and oxidation of the aluminum–magnesium alloy likely occur, which is reflected in the DSC curve showing a subsequent rise in temperature following an initial decrease, accompanied by a pronounced exothermic peak. Finally, in stage III (>985 °C), a minor exothermic peak is observed in the DSC curve, suggesting the oxidation exotherm of the remaining aluminum.

Figure 6d indicates that the heating rate significantly influences the variation in the thermal oxidation temperature curve. When the heating rate is lowered, the powders’ beginning reaction temperature and reaction process are moved forward, and the oxidation weight gain increases dramatically. As the heating rate increases, the initial reaction temperature of the powder also elevates. Schoenitz M.’s [41] investigation on the oxidation of aluminum powders at high heating rates also yielded similar results. This phenomenon is attributed to the rapid temperature rise, leading to a delayed increase in the powder’s temperature relative to the ambient conditions, consequently resulting in a higher initial reaction temperature. Furthermore, the amount of heat released in stage I under three different heating rates was calculated, as indicated by the DSC curves. The respective heat releases for heating rates of 10 K/min, 30 K/min, and 50 K/min were 51.64 J, 35.47 J, and 39.32 J, respectively. Based on the calculated results, it is found that, at a heating rate of 10 K/min, the heat release exhibited the highest concentration compared to heating rates of 30 K/min and 50 K/min in stage I. However, the degree of heat release concentration approached a similar level at heating rates of 30 K and 50 K.

### 3.2. Ignition Delay Time of Alloy Powders in Different Laser Flux Densities

**Figure 7:**
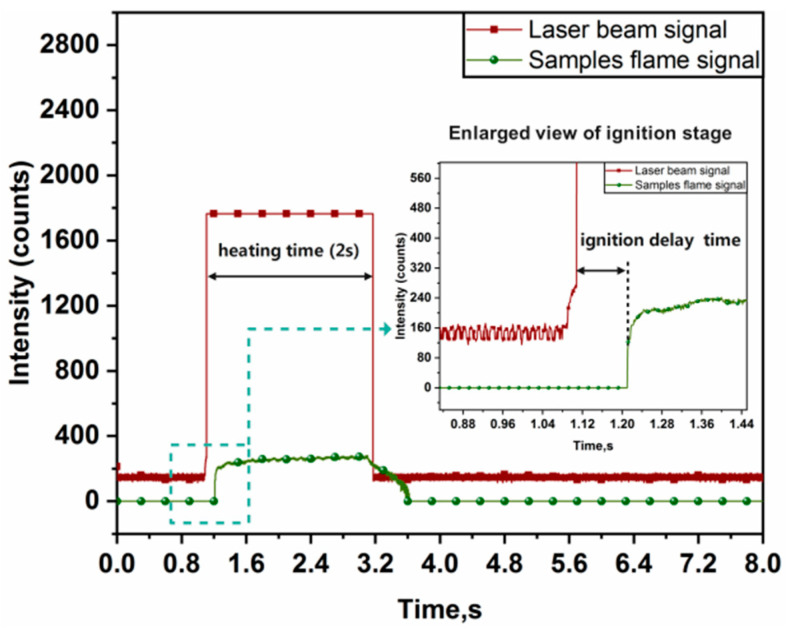
Typical spectral curve of laser ignition (laser flux density is 173.25 W/cm^2^).

**Figure 8:**
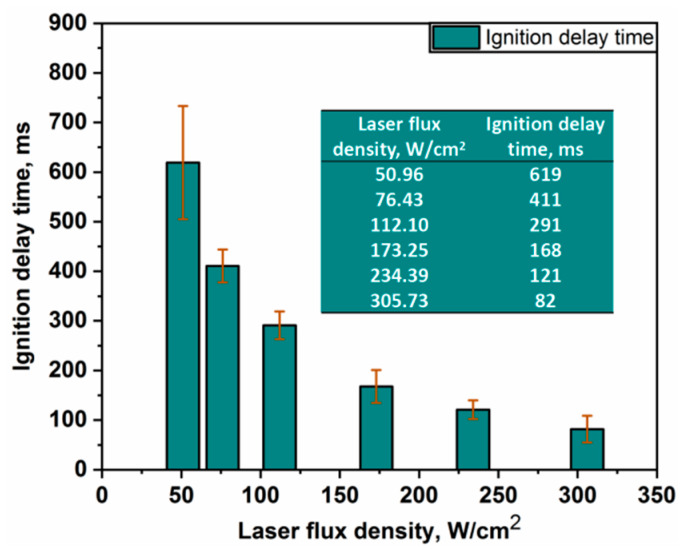
Average ignition delay times and their standard deviations at six laser flux densities.

**Figure 9:**
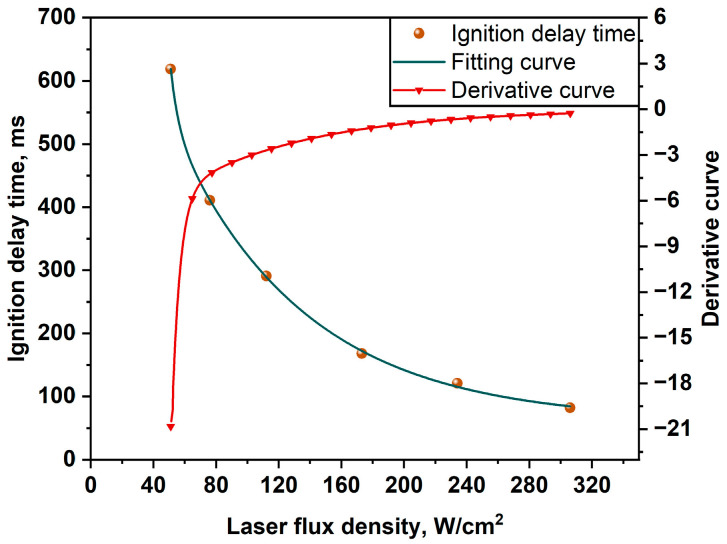
Average ignition delay time as a function of laser flux density.

Ignition experiments were performed at six laser ignition powers of 10 W, 15 W, 22 W, 34 W, 46 W, and 60 W, respectively, corresponding to the laser flux densities of 50.96 W/cm^2^, 76.43 W/cm^2^, 112.10 W/cm^2^, 173.25 W/cm^2^, 234.39 W/cm^2^, and 305.73 W/cm^2^. The representative spectral curve and the ignition delay time at a laser flux density of 173.25 W/cm^2^ are shown in Figure 7. The results of ignition delay times at six different experimental conditions are shown in Figure 8 and Figure 9.

From Figure 8, the ignition delay time of the Al-Mg-Li alloy powders was shortened significantly with the increase in laser flux density. These findings are explained by the faster heat accumulation caused by higher laser power, which raises the interior temperature. The lowered ignition delay time is no longer obvious if the ignition energy crosses a particular level. According to Figure 8, the ignition delay time was reduced by almost 87% when the laser flux density increased from 50.96 to 305.73 W/cm^2^. The average ignition delay times of the powder samples ranged from 619 to 291 ms at low power spectral density (50.96~112.10 W/cm^2^), and the ignition delay time drastically decreased with increasing laser flux density. The ignition delay time varied from 168 to 82 ms as the laser flux density increased (112.10~305.73 W/cm^2^). Besides, it should be noticed that in the low laser power situation, the ignition delay time inaccuracy is greater. The main reason for this result is that heat loss totally regulates the energy received by the sample stack, and the energy of the laser heating term and the heat loss term in the low-power scenario are unable to establish a stable equilibrium. This suggests that there is a crucial value (about 80 W/cm^2^) in the laser flux density range (50.96~305.73 W/cm^2^) where the laser ignition power has a significant impact on the ignition delay time. 

**Figure 10:**
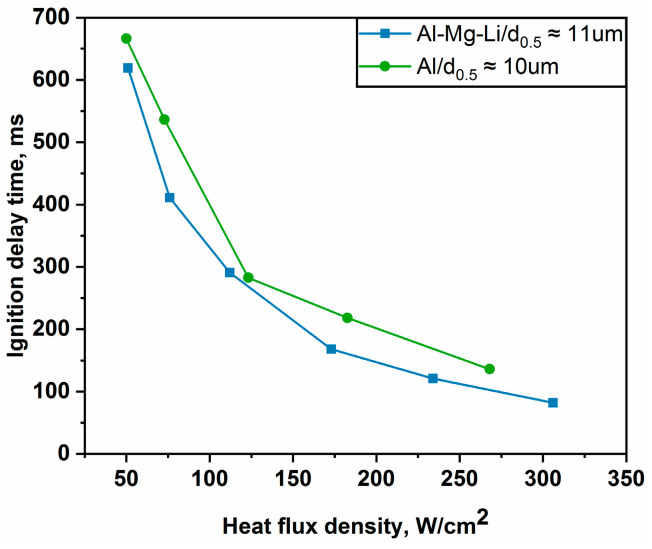
Comparison of ignition delay time between Al and Al-Mg-Li alloy powders.

Furthermore, the average ignition delay times are fitted using the least squares approach for six operating conditions. The fitting curve is shown in Figure 9. It can be seen that there is an obvious nonlinear relationship between the laser ignition delay time and the laser flux density of the alloy powder samples. The fitting curve is in good compliance with experimental results. Additionally, there is a mutation point in the derivative curve of Figure 9. This curve further indicates that the effect on the ignition delay time of Al-Mg-Li alloy powders is stronger at lower laser flux densities, whereas it becomes smaller at higher laser flux densities. Moreover, the same laser ignition system was used to perform a supplementary experimental study on Al powder with *d*_0.5_ of 10 um. The comparison of the ignition or delay times between pure Al and Al-Mg-Li alloy powders is shown in Figure 10. It can be seen that the ignition delay time of Al also decreases gradually when the laser power changes from low to high, which is similar to the change law of Al-Mg-Li. However, it is obvious that the ignition delay time of the Al-Mg-Li is lower than that of Al powders which share the approximate particle size. Based on the outcomes of laser ignition experiments involving Al-Mg-Li and pure Al, it was observed that the ignition delay times could be reduced by 4.6% to 26.9% within the heat flux density range of 50.96 to 305.73 W/cm^2^. These results indicate that Al-Mg-Li powders exhibit greater reactivity compared to pure aluminum powders. In the combustion chamber of a ramjet, it is imperative to note the extremely brief residence time of the fuel within the combustion chamber [8]. As a consequence, easy ignition of the fuel is a crucial requirement. The present study underscores the enhanced ignitability of Al-Mg-Li powders in comparison to pure Al powders, offering a more favorable option for ramjet applications.

In addition, similar to the minimum ignition energy (MIE) of dust clouds, we infer that metal powders also have an MIE. Previous studies have demonstrated that the MIE of dust clouds is related to the ambient temperature and the total heat flux [42,43,44]. In this work, the ignition delay time can be considered as the time accumulating the total heat flux to reach the MIE of the powder. Meanwhile, the ambient temperature is positively correlated to laser flux density, and as a result, the mathematical relationship between the ignition delay time and the laser flux density of Al-Mg-Li alloy powders can be described using the equation in Table 2. *E*_1_ and *E*_2_ listed in Table 1 were considered to be associated with the ignition energy of Li and Mg. The prediction equation was in good agreement with the experimental results, which shows that the equation has a certain reliability. The laser ignition delay time of Al-Mg-Li alloy powders can be predicted via the equation developed below at the laser flux density range of 50.96~305.73 W/cm^2^.

(1) $$y=A_{1}*\exp(-x/E_{1})+A_{2}*\exp(-x/E_{2})+y_{0}$$

The meanings of the letters in Table 1 are as follows:

1. *x*, the lase flux density, W/cm^2^;
2. *y*, the ignition delay time, ms;
3. *A*_1_ and *A*_2_, dimensionless coefficients;
4. *E*_1_ and *E*_2_, parameters related to ignition energy of Li and Mg;
5. y_0_, constant related to laser ignition delay time, ms;
6. *R*^2^, coefficient of determination, measuring the closeness of the model to the data points.

The interpretation of each letter in the above equation mainly refers to the Arrhenius equation:

(2) $$k=A\cdot exp(\frac{-Q}{RT})$$

where *k* is the rate constant for oxidation at a temperature of *T*, *A* is a constant the dimensions of which depend on units for *k*, *R* is the gas constant, and *Q* is the activation energy.

Kofstad, P.’s research [45] demonstrated that activation energies govern the oxidation of metals, and the oxidation reaction rate conforms to the Arrhenius formula (Equation (2)). In laser ignition tests, the energy *Q_l_* absorbed by the powder from the laser is a function of heat flux density (*x*), irradiated area (*s*), and irradiation time (*t*), expressed as follows:

*Q_l_* = *x* × *s* × *t* (3)

The alteration in temperature, ∆T, experienced by the powder during laser irradiation can be computed utilizing the laser irradiation energy (*Q_l_*), the material’s specific heat capacity (*c*), and its mass (*m*), as described by the following equation:

(4) $$∆T=\frac{Q_{l}}{c\cdot m}$$

Based on the above, the reaction rate constant *k* for the metal powder can be written as follows:

(5) $$k=A\cdot exp(\frac{-Q}{R\cdot (T_{0}+∆T)})=A\cdot exp(\frac{-Q}{R\cdot (T_{0}+\frac{Q_{/}}{c\cdot m})})$$

where $T_{0}$ is the initial temperature of the metal powder.

As known, the time necessary for the oxidation reaction exhibits an inverse relationship with the reaction rate constant. Hence, the oxidation duration of the metal powder may be construed as positively correlated with *k*^−1^, as follows:

(6) $$y\propto k^{-1}{=A}^{-1}\cdot exp(\frac{-(T_{0}\cdot c\cdot m)+x\cdot s\cdot t}{\frac{Q\cdot c\cdot m}{R}})\;$$

In Equation (6), $T_{0}$, *c*, *m*, *R* are all regarded as constants within the scope of this study. Consequently, the ignition delay time of the powder can be reasonably approximated as being proportional to the equation *A* × *exp*(*−x/E*), where E is associated with the activation energies of metals. The structure of Equation (1) aligns with this assertion. Given that the ignition tests utilized a singular type of powder and a limited range of laser ignition power, Equation (1) serves effectively as an empirical predictive model when applied within the heat flux density range of 50.96 to 305.73 W/cm^2^.

### 3.3. Ignition and Combustion Process at Different Laser Powers

**Figure 11:**
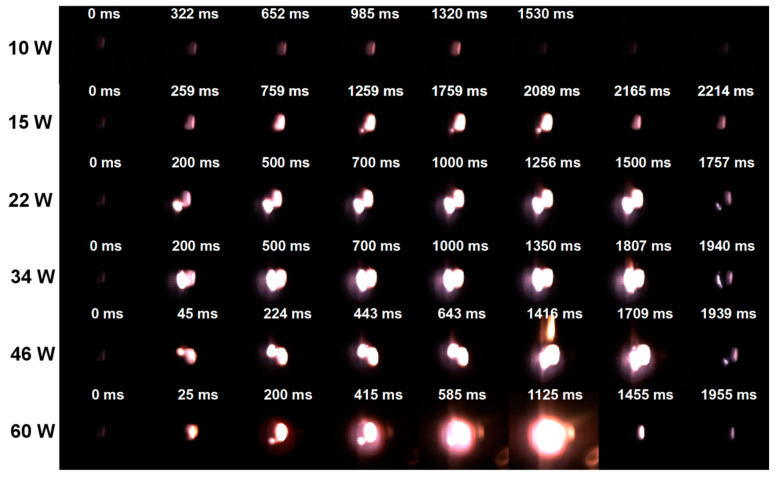
Ignition snapshots of Al-Mg-Li alloy powders at different laser powers.

The ignition process snapshots of powder samples at different ignition power are shown in Figure 11. Since the laser emission and the high-speed camera shot were not triggered simultaneously, the previous frame of the images where the light appeared was used as the 0 ms reference time in the snapshots.

It is evident that under various laser power irradiations, the ignition and combustion process of the powder samples photographed is quite different. The brightness of the combustion flame and the diameter range of the spot gradually grow when the laser power is increased. A jet flame appeared when the laser power was 46 W. The flame spot diameter and combustion intensity were both at their maximum with a laser power of 60 W. Additionally, it was discovered that the flames displayed a red gas phase profile at laser powers of 46 W and 60 W, demonstrating that the Li in the powder samples evaporated. Due to the rapid heating at high power, when the ignition power reached a certain number, the powder temperature surged quickly. The gas phase combustion then took place. The flames displayed the signature purplish-red flame color of Li.

Combining the TG-DSC thermal oxidation curves of the powder samples and the laser ignition pictures, the powder samples’ ignition and combustion processes may be classified into four stages: inert heating phase, partial combustion period, full combustion period, and quenching. The inert heating period begins when the CO_2_ laser is applied to the sample. During this time, the powder inside the crucible begins to absorb the laser energy and the temperature rises steadily. The powders are mostly heated during this phase and turn a carbon-red color. The powders start to melt as soon as the temperature reaches the components’ melting point. Meanwhile, a quick oxidation reaction took place. The local temperature of the powders keeps rising as the laser flux density increases and rapid thermal oxidation of the active metal components occurs. As the heat of the powders builds up quickly, local ignition and combustion occur. The powder sample starts to burn a great deal and enters the full combustion phase as a result of the dual action of the energy released from the igniting combustion and the laser irradiation energy. Later, the flame is extinguished because the condensed combustion products attach to the crucible and stop the metal powder in the bottom from further oxidizing and burning.

### 3.4. CCPs Characterization and Analysis

**Figure 12:**
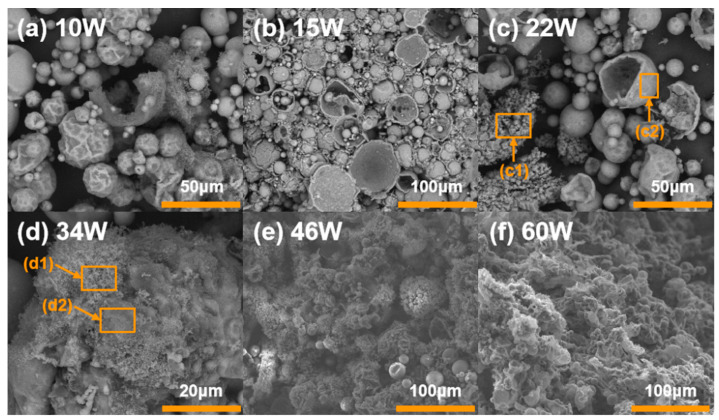
SEM images of condensed products at different laser powers.

**Figure 13:**
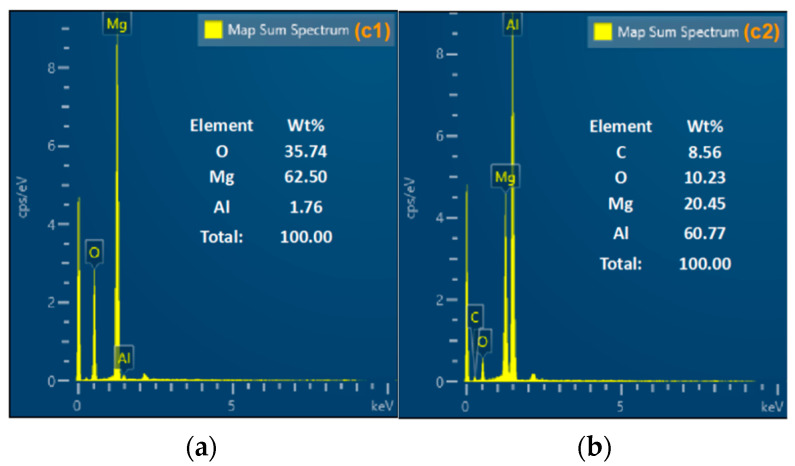
EDS images of combustion condensed products at 22 W, Figure 12c. (**a**) elemental distribution map of the c1 region; (**b**) elemental distribution map of the c2 region.

**Figure 14:**
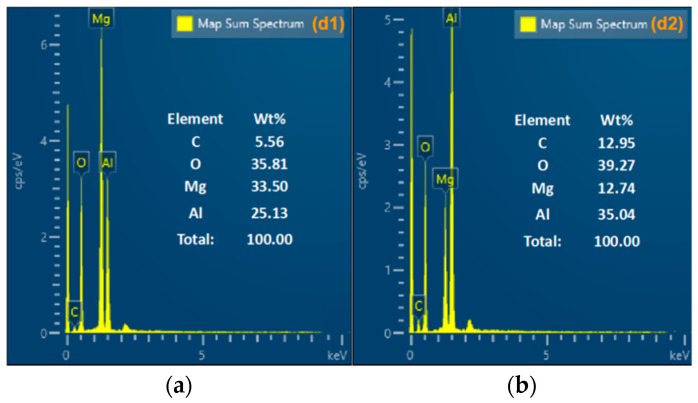
EDS images of combustion condensed products at 34 W, Figure 12d. (**a**) elemental distribution map of the d1 region; (**b**) elemental distribution map of the d2 region.

An SEM instrument was used to characterize the morphology of the condensed products in order to better investigate the ignition property of Al-Mg-Li powders at different laser ignition powders. SEM images of the products are shown in Figure 12. In addition, elemental composition analysis at laser powders of 22 W and 34 W are listed in Figure 13 and Figure 14 as representatives for characterizing two different combustion modes.

Compared with Figure 12a–f, the condensed phase products of different samples show different combustion characteristics. When the ignition power is low (10~22 W), a few of the powders maintain their original spherical shape, and there are also many particles with wrinkled surfaces, which is due to the melting of the internal core of the particle with a small number of particles ejected, leaving the oxide shell with a higher melting point. The metal oxide shell loses the core support and part of it deforms and collapses under the effect of the high temperature, and part of it is completely melted and ejected due to the high melting point, leaving only the oxide shell layer. Therefore, the SEM images show more spherical particles with folds and more obvious spherical oxide shells. Moreover, distinct oxide products and shell structure can be observed in Figure 12c at the laser power of 22 W. Elemental analysis of the spherical oxide shell layer listed in Figure 13 shows that the main components of the oxide shell layer are Mg, Al, and a small amount of O. It is thus clear that most of the metal elements in the oxide shell layer fail to undergo oxidation at this ignition power.

When the ignition power was higher (34~60 W), the product morphology changed significantly from spherical particles at low ignition power to continuous flocculent products at high ignition power. EDS analysis was carried out with the product represented at a laser power of 34 W. The analysis results are shown in Figure 14, which shows that the main components of the flocculent products located in Figure 14a were Mg, O, and Al with a content of 33.50, 35.81, and 25.13, respectively. In addition, the main components located in Figure 14b were Al, O, and Mg with a content of 35.04, 39.27, and 12.74, respectively. It can be seen that the reaction of the powder samples was more complete under the ignition power and the main components of the products were alumina and magnesia. 

### 3.5. Ignition Mechanism at Different Laser Powers

**Figure 15:**
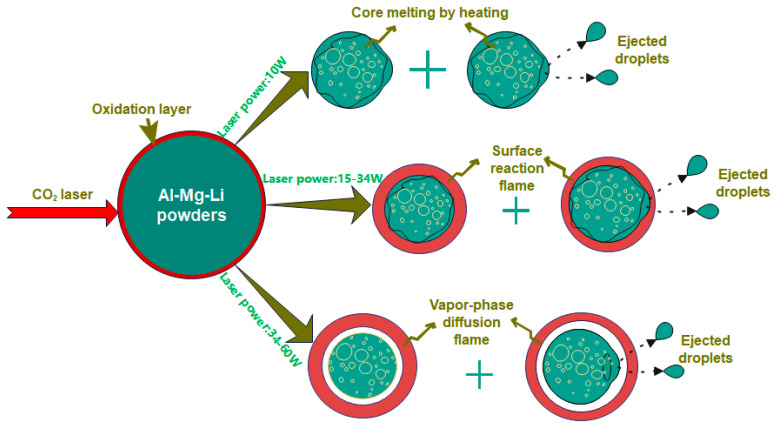
Ignition mechanism schematic at different laser powers.

It can be noticed that the ignition and combustion mechanism of the Al-Mg-Li alloy powders is different under multiple laser ignition powers considering the flame and condensed product morphology shown above. Figure 11 shows the flame shape at various laser powers. The fact that the powder can only be heated to a red color and has a very weak brightness at 10 W power is evident. In addition, it displays obvious ignition flames at 15~60 W of laser power. Additionally, the flame exhibits a clear gas phase profile at high power (34~60 W). Combining the results of Figure 11 and Figure 12, the ignition and combustion process of Al-Mg-Li can be described in Figure 15, referring to the combustion model of Al particles established by Tim Bazyn [46]. At low ignition power (10 W), the powder was heated to melt, accompanied by a small number of particles experiencing internal metal ejection. However, quenching occurred without successful ignition or combustion due to the low laser energy. When the ignition power was slightly higher (15~34 W), surface combustion occurred in the particles under the effect of the laser. In addition, due to the rapid accumulation of heat, some particles appeared to be burned with metal droplet jets. At higher laser powers (34~60 W), evaporation of metal components with low boiling points occurred and the particles exhibited vapor-phase diffusion combustion.

## 4. Conclusions

To investigate the thermal oxidation and ignition characteristics of Al-Mg-Li powders in an air atmosphere, TG-DSC tests were performed at three heating rates. Further, ignition and combustion characteristics of Al-Mg-Li, represented by flame shape and ignition delay times, were obtained using a laser ignition system at six laser powers. Finally, the ignition mechanism was illustrated by co-analysis of the flame snapshots and microstructure characterization of ignition products. The conclusions of this work are as follows:

1. The thermal oxidation behaviors of the powder samples were significantly correlated with the heating rates at different heating rates. At the lower heating rate of 10 K/min, the powder’s thermal hysteresis is less and the powder energy release in stage I is more concentrated. However, the degree of heat release concentration approached a similar level at the heating rates of 30 K and 50 K.
2. The ignition delay time reduces with increasing laser flux density, according to the results of the laser ignition tests. In addition, there is a clear nonlinear link between ignition delay time and laser flux density, which can be adequately described by the ExpDec2 model.
3. The ignition and combustion mode of Al-Mg-Li alloy powders changes from surface to gas phase combustion with the increase in ignition power. At low laser power (10 W), the powders are mostly heated only, with a small amount of active metal ejected outside, and the combustion flame is not obvious. At higher laser powers (15–34 W), the powders showed obvious combustion flame, accompanied by active metal droplet ejection, and the powders were considered to be mainly burning on the surface according to the product morphologies. At much higher laser power (34–60 W), the flame exhibits a clear gas phase profile and the microscopic morphology of the products is less likely to have a spherical shell. Therefore, it is considered that the powders mainly undergo gas-phase combustion with shell fragmentation.

According to previous reports [8], the ignition test of a powder ramjet engine has been successfully conducted utilizing Al powders as the fuel. This accomplishment establishes a foundational platform for the potential application of Al-Mg-Li powder in powder ramjets. This work shows that Al-Mg-Li alloys are easier to ignite than pure Al. During the ignition process, it was observed that Al-Mg-Li particles exhibit an active metal jet combustion mode. This phenomenon holds the potential to promote the ignition and combustion of the Al-Mg-Li powder. Furthermore, the relationship between the active metal injection phenomenon and the degree of promotion of ignition and combustion should be analyzed in detail. Additionally, in powder ramjets, the fuel is compacted rather than loose. Further investigations are necessary to simulate the actual state of fuel and provide more comprehensive information into the application of Al-Mg-Li alloys in powder ramjets.

## Figures and Tables

**Table 1:** The mass ratios of Al, Mg, and Li.

Element	wt%
Al	61.986%
Mg	30.846%
Li	9.768%

**Table 2:** The empirical formula of ignition delay time vs. laser flux density.

Values of Parameters					
*A*_1_	*A*_2_	*E*_1_	*E*_2_	*y*_0_	*R* ^2^
1.9 × 10^6^	857.6	5.1	84.6	61.5	0.998

## Data Availability

Data will be made available on request.
